# Death of Surgeon General Willard

**Published:** 1865-04

**Authors:** 


### DEATH OF SURGEON GENERAL WILLARD.

The following order has been received by Major-General R. L. Howard. The flag at the Arsenal is at half-mast to-day in accordance therewith:

GENERAL HEADQUARTERS, STATE OF NEW YORK,
Adjutant General's Office,
Albany, April 3d, 1865.

General Orders, No. 10.

The Commander-in-Chief is pained to announce the death of Brigadier-General S. D. Willard, Surgeon-General, which occurred on the 2d instant.

By his death the profession has been deprived of an eminent and devoted representative, the church of a consistent and upright member, the State of a pure and patriotic citizen.

Distinguished in his life by singleness of purpose, sincerity of conduct, and a wealth of scientific resources, he has left a good name, to be cherished as a noble heritage. His professional skill and energy were freely consecrated to the alleviation of the sufferings of the country's defenders, while his abilities were constantly employed in the advocacy of reform.

As a mark of respect for the memory of the deceased, and his distinguished position, it is ordered that the flags upon these Headquarters and the State Arsenals be displayed at half-mast on Wedesday, the 5th instant, from sunrise to sunset, and upon the different Regimental and Company Armories on the day next preceding the receipt of this order.

By order of the Commander-in-Chief.

J. B. STONEHOUSE, A. A. G.

The above notice of the death of Dr. Willard contains all that is known by us of the circumstances attending this sad event.

Dr. Willard has been Secretary of the State Medical Society for many years, and has contributed much, by his efforts in its behalf. He has also edited several works of local and historical value, and placed the profession under lasting obligations. Dr. Willard was a most pure, energetic, persevering and capable physician, and his early death will create a vacancy in the ranks of our profession, not easily supplied.

